# Protocol for spatial proteomic profiling of tonsil cancer microenvironments using multiplexed imaging-powered deep visual proteomics

**DOI:** 10.1016/j.xpro.2025.103901

**Published:** 2025-06-18

**Authors:** Xiang Zheng, Andreas Mund, Matthias Mann

**Affiliations:** 1 Novo Nordisk Foundation Center for Protein Research, University of Copenhagen, 2200 Copenhagen, Denmark; 2 Department of Biomedicine, Aarhus University, 8000 Aarhus, Denmark; 3 OmicVision Biosciences, BioInnovation Institute, 2200 Copenhagen, Denmark; 4 Proteomics and Signal Transduction, Max Planck Institute of Biochemistry, 82152 Martinsried, Germany

**Keywords:** Cancer, Microscopy, Biotechnology and bioengineering

## Abstract

Here, we present a protocol for spatial proteomic profiling of the tumor microenvironment in tonsil cancer using multiplexed imaging-powered deep visual proteomics (mipDVP). We describe steps for automated 22-plex immunofluorescence staining and imaging on formalin-fixed paraffin-embedded (FFPE) tissue sections, automated single-cell laser microdissection, and single-cell-type mass spectrometry. This workflow enables the spatially resolved isolation of distinct cell populations for proteomic analysis. We optimized this protocol for studying tumor-immune interactions, where it facilitates the systematic identification of biomarkers and functional cellular networks.

For complete details on the use and execution of this protocol, please refer to Zheng et al.^1^

## Highlights

• Protocol for spatial proteomics of FFPE tissues
• Steps for tumor microenvironment spatial analysis using 22-plex fluorescence imaging
• Instructions for single-cell isolation using automated laser microdissection
• Guidance on single-cell-type proteomic profiling using ultra-sensitive mass spectrometry

## Abstract (editor highlights)

Publisher’s note: Undertaking any experimental protocol requires adherence to local institutional guidelines for laboratory safety and ethics.

## Graphical abstract

**Figure:**
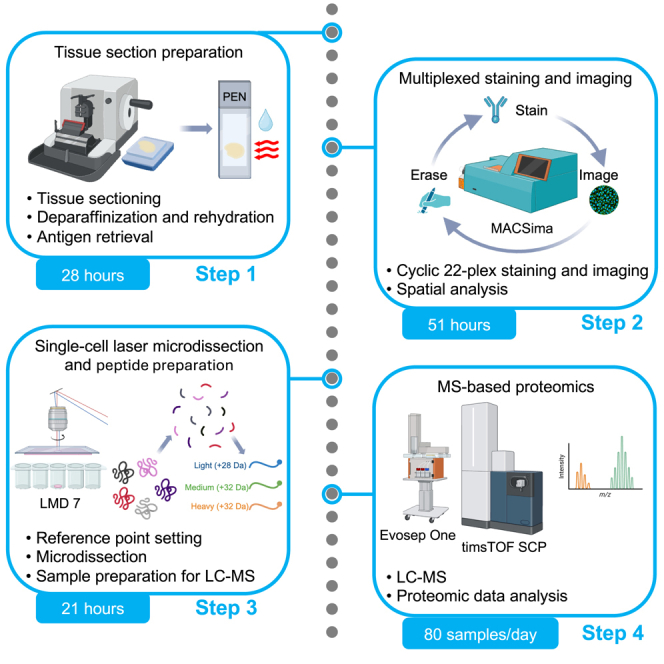

## Before you begin

### Protocol overview

This protocol presents multiplexed imaging-powered Deep Visual Proteomics (mipDVP), a spatially resolved workflow integrating cyclic 22-plex immunofluorescence imaging, automated laser microdissection, and high-sensitivity mass spectrometry to dissect the proteomic landscape of tonsil cancer microenvironments.^1^ The method subjects formalin-fixed paraffin embedded (FFPE) tissues to automated cyclic staining and imaging using the MACSima platform, combined with single-cell isolation using the Leica LMD7, thus enabling precise spatial mapping of tumor and immune cell populations. By coupling the Evosep One liquid chromatography (LC) system with the timsTOF SCP mass spectrometry, the method achieves deep proteome coverage from minimal input, capturing cell-type-specific functional states and intercellular interactions. Optimized for tumor-immune crosstalk, this reproducible framework bridges spatial biology with proteomic depth, offering insights into microenvironmental heterogeneity. While optimized for tonsil cancer, we have validated the protocol in colorectal cancer microenvironments.^1^

### Institutional permissions

Researchers must obtain ethics approval for human tissue use and secure informed consent for sample acquisition. We acquired all specimens—including human tonsil cancer FFPE tissues (see key resources table)—with informed consent and in accordance with standard protocols. In our case, we received approval from the relevant ethics committees, specifically ProteoGenex and Health Research Ethics Approval (VEK).

### Preparation

1. Antibody panel: Centrifuge the tubes of fluorescein isothiocyanate (FITC)/phycoerythrin (PE)/allophycocyanin (APC)-conjugated antibodies (Miltenyi Biotec, see key resources table) at 13,000 × g for 5 min at 22°C–26°C.

***Alternatives:*** FITC can be substituted with Vio B515, while Vio R667 may replace APC (spectrally similar).

***Note:*** Carefully pipette the supernatant to prevent potential aggregates that may cause staining artifacts.

***Note:*** Antibody staining optimization is Critical for robust cyclic immunofluorescence on the MACSima platform. Miltenyi optimizes its pre-conjugated antibodies (FITC, PE, APC) for photostability and cyclic imaging under standardized protocols. External vendor antibodies must match MACSima-compatible fluorophores and undergo validation in FFPE tissues. Key steps include testing specificity against single-plex controls or knockout tissues, screening for cross-reactivity in multiplex panels and titrating concentrations to balance signal-to-noise ratios. This validation process ensures compatibility with cyclic imaging, minimizes artifacts, and preserves spatial proteomic accuracy.

2. Microtome (HM 355S): Set the desired section thickness, check cooling if applicable, and perform a test section to verify smooth operation.

***Note:*** Before use, clean the microtome thoroughly, secure a sharp blade, and mount the specimen block parallel to the blade.

***Alternatives:*** Other rotary microtomes (e.g., Leica RM2255 or Thermo Fisher HM 325) may be used if they support equivalent section thickness precision (1–10 μm) and specimen cooling.

3. MACSima imaging platform (Miltenyi Biotec): Calibrate the system using control tissues to optimize multiplexed staining and imaging conditions.

***Alternatives:*** Our pilot data (n=3 colorectal cancer tissues) suggests cyclic immunofluorescence (CyCIF) with fluorophore bleaching (4.5% H_2_O_2_/24 mM NaOH in PBS, 1 hour at 22°C–26°C under white light)^2^ may substitute MACSima for ≤4-cycle staining (around 12-plex), but requires post-bleaching antibody revalidation and image registration tools (e.g., ASHLAR^3^) for alignment.

4. Laser microdissection system (LMD7, Leica): Align reference masks (.XML) to ensure precise laser microdissection for downstream analyses.

***Alternatives:*** The CellCut system (MMI) can substitute the Leica LMD7 for microdissection and collection of cell contours.^4^

5. Mass spectrometry setup: Configure the Evosep One LC system (Evosep) coupled to a timsTOF SCP mass spectrometer (Bruker) for proteomics.

***Note:*** Regular maintenance of both the LC and mass spectrometer, include cleaning the ion source and tuning the instrument to ensure optimal sensitivity and mass accuracy.

***Alternatives:*** The nanoLC systems (e.g., Thermo Ultimate 3000 or Vanquish UHPLC systems) can replace Evosep One if adjusted for low-flow (100 nL/min) gradients.

***Alternatives:*** The timsTOF SCP is replaceable with other high-sensitivity DIA-MS instruments (e.g., Orbitrap Astral) but requires setting re-optimization.^1^

6. Software installation: Install and configure these required programs: MACS iQ View, BIAS, DIA-NN, and FragPipe (see key resources table).

***Alternatives:*** For image analysis, open-source tools including DeepCell can substitute MACS iQ View for tasks including segmentation, though they lack native compatibility with MACSima's proprietary cyclic imaging data formats.^5^ For proteomics analysis, Spectronaut (Biognosys) may replace DIA-NN for DIA data processing but require manual parameter optimization and may yield slightly different peptide identification rates.

## Key resources table

**Table:** 

REAGENT or RESOURCE	SOURCE	IDENTIFIER
**Antibodies**		

Anti-CD3-APC	Miltenyi Biotec	130-120-269; RRID:AB_2876933
Anti-FOXP3-PE	Miltenyi Biotec	130-127-808; RRID:AB_2905207
Anti-Mast Cell Tryptase-APC	Miltenyi Biotec	130-125-278; RRID:AB_2857771
Anti-PD1-PE	Miltenyi Biotec	130-117-384; RRID:AB_2727929
Anti-CD107a-APC	Miltenyi Biotec	130-126-203; RRID:AB_2889419
Anti-CD38-PE	Miltenyi Biotec	130-126-438; RRID:AB_2904976
Anti-HLA-DR-FITC	Miltenyi Biotec	130-123-076; RRID:AB_2857572
Anti-CD4-PE	Miltenyi Biotec	130-127-906; RRID:AB_2921815
Anti-CD45RA-APC	Miltenyi Biotec	130-112-097; RRID:AB_2819365
Anti-CD20-FITC	Miltenyi Biotec	130-118-292; RRID:AB_2857425
Anti-CD8-PE	Miltenyi Biotec	130-117-201; RRID:AB_2857415
Anti-Ki67-FITC	Miltenyi Biotec	130-117-691; RRID:AB_2733585
Anti-CD45RO-APC	Miltenyi Biotec	130-113-556; RRID:AB_2733381
Anti-Cytokeratin-FITC	Miltenyi Biotec	130-112-743; RRID:AB_2651496
Anti-CD57-PE	Miltenyi Biotec	130-111-810; RRID:AB_2658748
Anti-CD11b-PE	Miltenyi Biotec	130-128-773; RRID:AB_2904691
Anti-Podoplanin-APC	Miltenyi Biotec	130-126-165; RRID:AB_2905370
Anti-CD68-PE	Miltenyi Biotec	130-128-345; RRID:AB_2905104
Anti-Vimentin-FITC	Miltenyi Biotec	130-116-508; RRID:AB_2727581
Anti-SMA-FITC	Miltenyi Biotec	130-123-363; RRID:AB_2857589
Anti-CD31-PE	Miltenyi Biotec	130-128-769; RRID:AB_2904938

**Biological samples**		

FFPE human tonsil cancer	ProteoGenex	#652323T2(3)

**Chemicals, peptides, and recombinant proteins**		

Acetone	Sigma-Aldrich	270725
VECTABOND	Vector Laboratories	SP-1800-7
Xylene	Carl Roth	4436.1
Ethanol	Merck	1070172511
Ethylenediaminetetraacetic acid (EDTA) buffer	Merck	E1161
Glycerol	Merck	G7757
Triethylammonium bicarbonate	Merck	T7408
n-dodecyl-β-D-maltoside	Merck	850520P
Acetonitrile	Thermo Fisher Scientific	A955-1
LysC	Promega	VA1170
Trypsin	Promega	V5280
Trifluoroacetic acid	VWR	153112E
Formic acid	Merck	5.33002
Formaldehyde, CH_2_O	Sigma-Aldrich	252549
Formaldehyde, CD_2_O	Sigma-Aldrich	492620
Formaldehyde, ^13^CD_2_O	Sigma-Aldrich	596388
Sodium cyanoborohydride	Sigma-Aldrich	8180530025
Ammonia	Merck	5.33003
LC-MS-grade water	VWR	83645.290
2-propanol	Merck	1027811000

**Deposited data**		

Mass spectrometry proteomics data	Zheng et al.^1^	PRIDE: PXD057118
MACSima imaging data	Zheng et al.^1^	BioImage Archive: S-BIAD1116
Antibody worksheet (Document S1)	Zheng et al.	Mendeley Data https://doi.org/10.17632/5bnrpkwkx9.1
FASTA file (Document S2)	Zheng et al.	Mendeley Data https://doi.org/10.17632/5bnrpkwkx9.1

**Software and algorithms**		

MACS iQ View software v.1.2.2	Kinkhabwala et al.^6^	https://www.miltenyibiotec.com/DK-en/products/macs-iq-view-spatial-biology-software.html
Biology Image Analysis (BIAS) software v.1.3.0	Mund et al.^7^	https://single-cell-technologies.com/bias-2/
LMD software v.8.3	Mund et al.^7^	https://www.leica-microsystems.com/products/microscope-software/p/leica-lmd-software/
DIA-NN v.1.8.1	Demichev et al.^8^	https://github.com/vdemichev/DiaNN
FragPipe v.18.0	Kong et al.^9^	https://fragpipe.nesvilab.org/
BioRender	BioRender	www.biorender.com

**Other**		

HM 355S microtome	Fisher Scientific	23-900-672
MACSima imaging platform	Miltenyi Biotec	MACSima
LMD7 laser microdissection system	Leica Microsystems	LMD7
Evosep One LC system	Evosep	Evosep One
timsTOF SCP	Bruker	timsTOF SCP
SpeedVac	Merck	EP5305000169
NanoDrop One spectrophotometer	Fisher Scientific	13-400-525
Membrane PEN slides	Zeiss	415190-9041-000
MACSima two-well imaging frame	Miltenyi Biotec	130-124-675
MACSima deepwell plates	Miltenyi Biotec	130-126-865
MACSima running buffer	Miltenyi Biotec	130-121-565
H&E stain kit	Abcam	ab245880
Biorupter	Diagenode	B01020001
Thermal cycler	Bio-Rad	S1000
Aurora Elite CSI third-generation column	IonOpticks	AUR3-15075C18-CSI

## Materials and equipment

**Table:** Antigen retrieval buffer

Reagent	Stock concentration	Volume added	Final concentration
EDTA buffer	10×	10 mL	1×
Glycerol	100%	10 mL	10%
Distilled deionized water (ddH_2_O)	-	80 mL	-
Total	-	100 mL	-

***Note:*** Prepare freshly before use.

**Table:** Lysis buffer

Reagent	Stock concentration	Volume added	Final concentration
Triethylammonium bicarbonate (TEAB)	1 M	60 μL	60 mM
LC-MS-grade water	-	940 μL	-
Total	-	1000 μL	-

***Note:*** Use only LC-MS-grade water to avoid contaminants. Prepare fresh before use.

***Note:*** To prepare the lysis buffer with 0.01% n-dodecyl-β-D-maltoside (DDM), add 10 μL of 1% DDM stock solution to a clean container with 930 μL of LC-MS-grade water. Then, add 60 μL of 1 M TEAB in a fume hood. Prepare freshly before use.

**CRITICAL:** Both DDM and TEAB may irritate the skin, eyes, and respiratory tract. Handle these chemicals with appropriate personal protective equipment (PPE), including gloves, goggles, and protective clothing, and work in a fume hood.

**Table:** Buffer A

Reagent	Stock concentration	Volume added	Final concentration
Formic acid (LC-MS grade)	100%	1 mL	0.1% (v/v)
LC-MS-grade water	-	999 mL	-
Total	-	1000 mL	-

***Note:*** Store in a glass bottle at 22°C–26°C. Stable for 3 months when properly sealed.

**CRITICAL:** Formic acid is corrosive and can cause burns. Handle it carefully with appropriate PPE and work in a fume hood.

**Table:** Buffer B

Reagent	Stock concentration	Volume Added	Final concentration
Formic acid (LC-MS grade)	100%	1 mL	0.1% (v/v)
Acetonitrile (LC-MS grade)	100%	999 mL	99.9% (v/v)
Total	-	1000 mL	-

***Note:*** Store in a glass bottle at 22°C–26°C. Stable for 6 months when properly sealed.

**CRITICAL:** Acetonitrile is flammable and toxic. Handle in a fume hood with appropriate PPE. Store in a flammable cabinet away from heat and ignition sources.

### High-performance computer setup

The analysis pipeline requires a high-end workstation. Compatible operating systems include Windows 10 (64-bit, version 1703 or later), macOS 10.14.6 or later, and Linux. We currently suggest the following minimal specifications: a processor with at least 8 cores (e.g., Intel Core i5, AMD Ryzen 3, or Apple M1) and 32 GB RAM (16 GB for macOS). Storage should include a 2 TB or larger M.2/NVMe SSD, with external SSDs connected via USB 3.0 or Thunderbolt for additional space. We prefer a 4K UHD display (3840 x 2160) or higher for working with high-resolution imaging data. We also recommend dedicated graphics cards from Nvidia or AMD with 8 GB vRAM or more, supporting Metal (macOS) or tessellation (Windows). For large dataset transfers and processing, we suggest a high-speed network connection, such as 10 Gbit LAN. Validated systems include the HP Z4 G4 workstation, HP ZBook Fury 15 G7 laptop, and recent Apple systems like the MacBook Pro or iMac Pro.

## Step-by-step method details

### Tissue section preparation and antigen retrieval for multiplexed staining and imaging

**Timing: 28 h**

This step describes the preparation of FFPE tissue sections, including deparaffinization, rehydration, and antigen retrieval to optimize sample compatibility with multiplexed cyclic immunofluorescence imaging.

1. Pre-treat polyethylene naphthalate (PEN) membrane slides.
  a. Expose PEN slides to UV light for 1 h.
  b. Immerse slides in acetone for 5 min.
  c. Immerse slides in Vectabond (1:50 diluted in acetone) for 5 min.
  d. Rinse in distilled water for 30 seconds.
  e. Air-dry 12 to 18 h.

**CRITICAL:** Acetone is highly flammable and can irritate skin and eyes. Store in a flammable cabinet away from ignition sources and handle in a fume hood. Vectabond is an irritant, handle in a fume hood with appropriate PPE.

**Figure 1:**
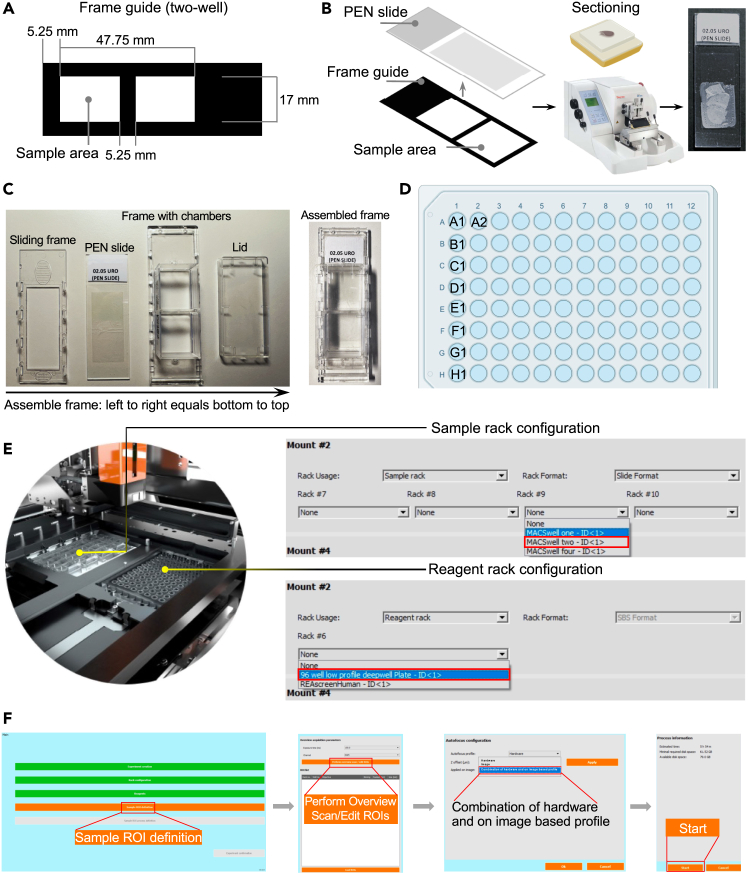
Sample preparation workflow for MACSima imaging system (A) Sample alignment guide showing the sectioning area compatible with the two-well imaging frame. (B) Guide for tissue sectioning onto PEN membrane slides with sections aligned to imaging frame wells. (C) Assembly of the two-well imaging frame, designed to secure tissue sections while enabling reagent access during automated staining cycles. (D) Deepwell plate (96-well format) preloaded with fluorescently conjugated antibodies for automated dispensing. (E) System rack setup, showing the imaging frame and deepwell plate. (F) Sample regions of interest (ROI) and autofocus configuration. The screenshots originate from the interface of MACSima Imaging System (#130-121-164 ) with software v.0.13.0.
  c. Bake slides at 37°C 12 to 18 h.

***Note:*** Miltenyi Biotec 130-124-675 kit (Key resources table) provides the frame guide Figures 1A and 1B).

2. Section FFPE tissue.
  a. Cut 3 μm tissue sections.
  b. Mount tissue sections in the lower half of PEN slides, within the rectangular guide area (Figures 1A and 1B).

3. Deparaffinize and rehydrate.
  a. Bake slides at 60°C for 20 min.
  b. Immerse slides sequentially in:
    i. Xylene: 3 cycles of 5 min each.
    ii. 100% Ethanol: 2 cycles of 2 min each.
    iii. 95% Ethanol: 2 min.
    iv. 80% Ethanol: 2 min.
    v. 70% Ethanol: 2 min.
    vi. ddH_2_O: 2 min.

**CRITICAL:** Xylene is toxic, flammable, harmful via inhalation, ingestion, or skin absorption. Ethanol is flammable and irritates on contact or inhalation. Store both in flammable cabinet away from ignition. Use in fume hood.

**CRITICAL:** Ensure the slides remain hydrated after deparaffinization to prevent non-specific antibody binding.

4. Heat-induced epitope retrieval and prepare slides for multiplexed staining (Troubleshooting).
  a. Heat slides in EDTA buffer with 10% glycerol at 88°C in a water bath for 45 min.
  b. Mount slides in the MACSima two-well frame (Key resources table; Figure 1C).
  c. Add 550 μL running buffer (Miltenyi Biotec 130-121-565).
  d. Incubate the sample with a 1:5 (1 μg/mL) dilution of DAPI staining solution (Miltenyi Biotec 130-127-574) in the dark at 22°C–26°C for 10 min.
  e. Wash the slides three times with running buffer.
  f. Add 550 μL of running buffer before proceeding to the staining cycles.

**CRITICAL:** Adding 10% glycerol to the EDTA buffer is crucial for preserving the integrity of PEN slides. Ensure slides are fully submerged to prevent uneven antigen retrieval.

**Pause Point:** Store slides in running buffer at 4°C for ≤1 week.

### Automated 22-plex cyclic immunofluorescences staining and imaging

**Timing: 51 h**

***Note:*** Conduct autofluorescence photobleaching in the FITC, PE, and APC channels as cycle 0 before the staining cycles begin

This step details the cyclic staining of FFPE tissues using FITC/PE/APC-conjugated antibodies and high-resolution imaging with the MACSima platform, enabling spatial characterization of cellular marker expression.

5. Complete the antibody worksheet (Document S1; see key resources table).
  a. Provide background information about the experiment (Document S1 Tab 1_ Experimental Setup).
  b. Define the plate layout as a two-well format by marking the frame area with an ‘X’ (Document S1 Tab 1_ Experimental Setup).
  c. Specify the exposure times for the respective fluorochromes:
    i. 6, 36, and 216 ms for the APC channel.
    ii. 5, 20, and 80 ms for the PE channel.
    iii. 8, 32, and 128 ms for the FITC channel (Tab 1_ Experimental Setup).
  d. Fill out the antibody worksheets (Document S1 Tab 3-5_ConjugatesPE/FITC/APC).
  e. Click ‘Fill list’ (Document S1 Tab 2_ConjugatesAll) to automatically retrieve information from Tabs 3–5.
  f. Review the final antibody sequence, then export the entire file as an .xlsx.
6. Dispense antibodies conjugated with FITC, PE, or APC into a MACSima deepwell plate (Key resources table; Table 1; Figure 1D; Troubleshooting).

**Table 1:** Antibody dispensing overview

Staining cycle	Deep well destination	Antigen name	Antibody clone	Fluorochrome	Antibody dilution factor	Antibody (μL)	FcR blocking buffer (μL)	DAPI (μL)	Running buffer (μL)
Cycle 1	A1	CD3	REA1151	APC	25	22	11	0	475
		FoxP3	REA1253	PE	13	42			
Cycle 2	B1	Mast Cell Tryptase	REAL798	APC	50	11	0	0	497
		PD1	PD1.3.1.3	PE	13	42			
Cycle 3	C1	CD107a	REAL653	APC	50	11	0	11	517
		CD38	REAL719	PE	25	22			
Cycle 4	D1	CD45RA	REAL164	APC	25	22	11	0	484
		HLA-DR	REAL550	FITC	50	11			
		CD4	REA1307	PE	25	22			
Cycle 5	E1	CD45RO	REA611	APC	25	22	0	0	495
		CD20	REA1087	FITC	50	11			
		CD8a	REA1024	PE	25	22			
Cycle 6	F1	Ki-67	REA183	FITC	50	11	0	11	528
		CD57	REA769	PE	50	11			
Cycle 7	G1	Cytokeratin	REA831	FITC	50	11	11	0	517
		CD11b	REA1321	PE	50	11			
Cycle 8	H1	Podoplanin	REA446	APC	50	11	0	0	523
		Vimentin	REA409	FITC	50	11			
		CD68	REA1306	PE	100	6			
Cycle 9	A2	SMA	REAL650	FITC	100	6	0	11	512
		CD31	REA1312	PE	25	22

***Alternatives:*** For researchers who prefer pre-validated antibody panels, commercially available options such as the REAplex Immuno-oncology Core (20 antibody panel; Miltenyi Biotec, 130-136-302) and REAscreen Immuno-oncology Kit (61 antibody panel; Miltenyi Biotec, 130-132-525) can reduce optimization time for multiplexed imaging.

**CRITICAL:** Use antibodies conjugated with FITC, PE, and APC fluorochromes due to their relatively low photostability. Since the DAPI signal is crucial for image preprocessing, it is advisable to retain DAPI every third or fourth cycle to ensure consistent alignment.

7. Set up the reagent and sample racks and specify the region of interest.
  a. Begin by selecting ‘Experiment Creation’ in the interface of MACSima Imaging System (Model #130-121-164) with software v.0.13.0.
  b. Enter the experiment information and import the antibody worksheet (Datasheet S1).
  c. Choose ‘MACSwell Two’ for sample rack configuration (Figure 1E).
  d. Select ‘96-Well Low-Profile Deepwell Plate’ for reagent rack configuration (Figure 1E).
  e. Insert the framed slide and deepwell plate into the MACSima system.
  f. Define the region of interest (ROI) based on the DAPI signal by selecting ‘Sample ROI Definition’.
  g. Click on ‘Perform Overview Scan/Edit ROIs’ and select the desired ROI (Figure 1F).
    ***Note:*** Click on the image and drag to define a ROI. Release the mouse button to finish.
  h. Select ‘Edit Autofocus Profile’ and choose ‘Combination of Hardware-Based and Image-Based Profile’ from the ‘Autofocus Configuration’ options (Figure 1F).
  i. Click ‘Start’ to initiate the experiment (Figure 1F).
8. Upon completion, a summary screen will appear. Click ‘Open’ to retrieve the data, including ‘Calibration’, ‘ExperimentDefinition’, ‘PreprocessedData’ and ‘RawData’.

***Optional:*** After multiplexed staining and imaging, apply auxiliary Hematoxylin and Eosin (H&E) staining (H&E stain kit, Key resources table) to PEN slides for enhanced tissue visualization and precise mask alignment for laser microdissection. Cover the tissue with Mayer’s Hematoxylin and incubate for 5 min, then rinse twice in distilled water. Apply Bluing Reagent for 10–15 seconds, rinse again, dip in absolute alcohol, and stain with Eosin Y for 2–3 min. Rinse with absolute alcohol and dry the slide at 37°C 12 to 18 h.

***Alternatives:*** For DAPI-based mask alignment, rinse the slides with ddH_2_O for 1 minute prior to drying for microdissection.

**Pause Point:** Store slides dry at 22°C–26°C until image analysis is complete and ready for laser microdissection.

### Image preprocessing, segmentation, classification, and spatial analysis

**Timing: 4–26 h (scales with antibody panel size, number of classified cell populations, and spatial feature complexity)**

This step explains image preprocessing, cell segmentation, and the spatial classification of distinct cell types to define their proximity relationships within the tumor microenvironment.

**Figure 2:**
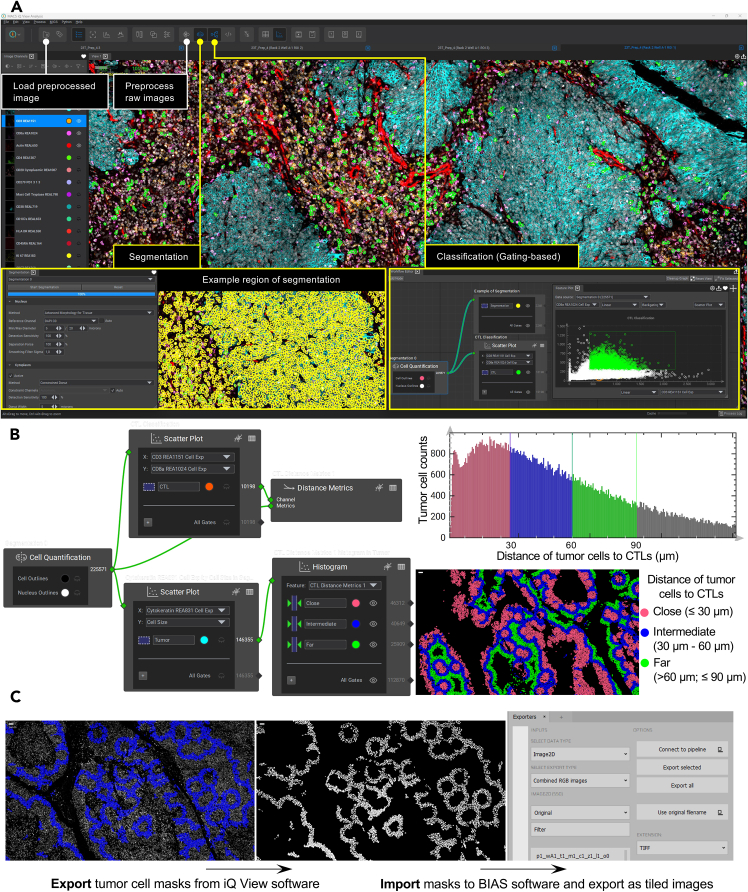
Image analysis workflow for spatial characterization (A) MACS iQ View software (v.1.2.2) interface highlighting key modules: image preprocessing tools, processed image loading, and interactive panels for segmentation and gating-based classification. Example regions demonstrate cell segmentation (yellow masks) and immune cell gating (cytotoxic T cells (CTLs), green masks). (B) Spatial analysis workflow categorizing tumor cells into three proximity zones relative to cytotoxic T cells: close (≤30 μm), intermediate (30–60 μm), and far (>60 μm; ≤90 μm). (C) Integration of MACS iQ View and BIAS software for mask alignment of downstream laser microdissection. Export masks of phenotypically defined cells (tumor cells with intermediate distance to CTLs) from iQ View and import them into BIAS for reference point set up. Scale bars: 30 μm.
  b. During the preprocessing setup, select the ‘Nikon 20x Plan Flour ELWD’ lens, set the exposure to ‘Automatic’ at 100% scaling, and enable the ‘Crop to DAPI’ option.
  c. Click ‘Start Preprocessing’ to initiate the process.
  d. Once preprocessing is complete, load the preprocessed image into iQ View software by selecting the ‘Project Browser’ icon (Figure 2A).

***Note:*** The preprocessing pipeline stitches images from one ROI, registers them and subtracts the background signal.

***Note:*** The cache system accelerates image data accessibility without overburdening the computer’s memory. We recommend using an SSD dedicated to cache storage, as HDDs are significantly slower in comparison.

9. Preprocess images.
  a. Load ‘RawData’ into MACS iQ View software (v.1.2.2) by selecting the ‘Open Raw Directory’ option under the ‘Preprocessing’ icon (Figure 2A).

10. Conduct cellular segmentation using nuclei and cell membrane markers (Troubleshooting).
  a. In segmentation panel, select the ‘Advanced Morphology for Tissue’ method with DAPI as the reference channel for nucleus segmentation.
  b. In segmentation panel, select the ‘Constrained Donut’ method with automatic constraint channels for cytoplasm segmentation (Figure 2A).
11. Conduct cellular classification using a gating-based strategy, identifying distinct cell types and subtypes. These can include:
  a. Cytokeratin+ tumor cells.
  b. CD3+CD8+ cytotoxic T cells (CTL), with subtypes CD3+CD8+CD45RA–CD45RO–, CD3+CD8+CD45RA+CD45RO+, CD3+CD8+CD45RA+CD45RO–, and CD3+CD4+CD45RA–CD45RO+.
  c. CD3+CD4+ helper T cells, with subtypes CD3+CD4+CD45RA–CD45RO–, CD3+CD4+CD45RA+CD45RO+, CD3+CD4+CD45RA+CD45RO–, and CD3+CD4+CD45RA–CD45RO+.
  d. CD20+CD3–CD38– B cells, with subtypes CD20+CD3–CD38–CD45RA+CD45RO+, CD20+CD3–CD38–CD45RA–CD45RO–, CD20+CD3–CD38–CD45RA+CD45RO–, and CD20+CD3–CD38–CD45RA–CD45RO+.
  e. CD38+CD20–Ki67– plasma cells.
  f. CD57+CD107a+HLA–DR+ NK cells.
  g. CD68+HLA–DR+ macrophages.
  h. Mast Cell Tryptase+ mast cells.
  i. Podoplanin+ lymphatic endothelial cells.
  j. CD31+ blood vessel endothelial cells.
12. Spatial distance analysis.
  a. In the ‘Workflow Editor’ panel, create distance metrics for a classified cell type (e.g., CTLs) (Figure 2A).
  b. In ‘Feature Plot’ panel, plot histograms to visualize the distribution of tumor cells at various distances from CTLs.

***Note:*** In our analysis of tumor-immune spatial interactions (Figure 2B), we classified tumor cell distances from CTLs into three distance categories: close proximity ≤30 μm, intermediate distances 30 μm–60 μm, and far away >60 μm and ≤90 μm.^1^

### Exporting and aligning masks for laser microdissection

**Timing: 20 min**

This step involves generating and aligning cell-specific segmentation masks for precise laser microdissection of spatially resolved cell populations.

13. Export masks and corresponding ROI images (including the DAPI channel) from MACS iQ View software and import them into BIAS software (v.1.3.0)^7^ for spatial registration (Figure 2C; Troubleshooting).
  ***Note:*** This is to align cell-of-interest masks with the membrane section for downstream laser microdissection.
  a. From BIAS, export masks as tiled images and save them in a dedicated folder (Figure 2C).
  b. Load the DAPI channel reference image into BIAS.
  c. Navigate to the ‘Segmentation’ module and select ‘Bulk Load Segmentation’.
  d. Configure ‘input type’ as ‘Binary images’.
  e. Establish three reference points (e.g., tissue landmarks or alignment markers) in BIAS.
  f. Export mask coordinates as XML files using the ‘greedy’ sorting method.
  g. Import these XML files into Leica LMD software (v.8.3) for precise alignment and automated microdissection.

### Laser microdissection of spatially defined cell populations

**Timing: 20–50 min per sample**

This step provides the settings and workflow for isolating targeted cell populations for downstream proteomic analysis.

14. Microdissect cells (Troubleshooting).
  a. Perform laser microdissection using Leica LMD7 with the following settings:
    i. Magnification: 63x (HC PL FLUOTAR L ×63/0.70 objective).
    ii. Cutting method: middle pulse.
    iii. Aperture: 1.
    iv. Cutting speed: 25.
    v. Middle pulse count: 4.
    vi. Final pulse: 1.
    vii. Laser power: 35.
    viii. Head current: 50%.
    ix. Pulse frequency: 2,600 Hz.
  b. Microdissect each cell type of interest into 3 or 4 wells of a 384-well plate (serving as technical triplicates or quadruplicates) from the same membrane section.
    ***Note:*** Standardize the microdissected material per well by ensuring a consistent total surface area, for instance, to 37,000 μm^2^.^1^
  c. Following microdissection, add 40 μL 100% acetonitrile.
  d. Centrifuge the 384-well plate at 2,000 × g for 10 min.
  e. SpeedVac the samples until completely dry.
    ***Note:*** Omit the outermost rows and columns of the 384-well plate due to LMD stage constraints. Pellet cells with 100% acetonitrile before vacuum drying.
    **Pause Point:** Store the well-covered 384-well plate dry at 22°C–26°C for ≤2 week.

### Proteomic sample preparation for mass spectrometry

**Timing: 33 h**

This step outlines enzymatic digestion, peptide labeling, and sample cleanup for proteomic analysis, ensuring high-quality inputs for mass spectrometry.

15. Reference channel sample (Δ0 sample) preparation for LC-MS analysis.
  a. Scrape deparaffinized tissue samples from membranes and transfer them into 1.5 mL Eppendorf tubes.
  b. Add 100 μL of lysis buffer (60 mM TEAB with 0.01% DDM) to each tube.
  c. Spin down the tubes and incubate for 1 hour at 95°C in a thermal cycler or shaker (800 × g).
  d. After incubation, centrifuge at 1,000 × g for 1 minute.
  e. Sonicate the samples using a Diagenode Bioruptor 300 (30 seconds on/off for 12 cycles at high power).
    ***Note:*** Probe sonication is not recommended due to localized overheating and disruptive shear forces that compromise antigen integrity for downstream mass spectrometry.
  f. Add 100% acetonitrile to achieve a final concentration of 12.5%.
  g. Shake for 10 seconds, then spin down at 1,000 × g for 1 minute.
  h. Incubate the tubes for 1 hour at 75°C in a thermal cycler (800 × g), then cool to 22°C–26°C.
  i. Measure protein concentrations in each sample using a NanoDrop One spectrophotometer with A280 absorbance.
    ***Alternatives:*** Protein quantification can alternatively use Qubit fluorometric assay or BCA assay, but avoid Bradford assays due to DDM incompatibility.
  j. Dilute LysC and trypsin to 1:50 enzyme-to-protein ratio using a buffer composed of 60 mM TEAB and 12% acetonitrile based on protein concentration.
  k. Add the appropriate amounts of enzymes to each sample and incubate 12 to 18 h at 37°C, shaking at 300 rpm.
  l. Acidify the digested peptides with 1% trifluoroacetic acid (TFA).
  m. SpeedVac the samples until completely dry.
  n. Reconstitute the samples in 400 μL of Buffer A (0.1% formic acid).
16. Reference channel sample (Δ0 sample) peptide cleanup (desalting) using C18 columns.
  a. Activate C18 columns with **100 μL methanol** and centrifuge at 750 × g for 5 min.
  b. Equilibrate C18 columns with 100 μL of Buffer B and centrifuge at 750 × g for 5 min.
  c. Add 100 μL of Buffer A and centrifuge at 750 × g for 5 min.
  d. Load samples and wash C18 columns twice with Buffer A.
  e. Elute peptides with 100–500 μL of 80% acetonitrile with 0.1% formic acid.
  f. SpeedVac the samples until completely dry.
  g. Reconstitute peptides to 0.125 μg/μL in 60 mM TEAB with 10% acetonitrile (pH 8.5).
17. Reference channel sample (Δ0 sample) light dimethyl labeling.^10^
  a. Add light formaldehyde (CH_2_O) to each sample to achieve a final concentration of 0.15%.
  b. Add light sodium cyanoborohydride (NaBH_3_CN) to achieve a final concentration of 0.023 M.
  c. Incubate the sealed plate at 22°C–26°C for 1 hour.
  d. Add ammonia to quench the reaction (final concentration: 0.13%).
  e. Spin down at 1,000 × g for 1 minute, then incubate for 2 min.
  f. Acidify the peptides with 10% TFA to achieve a final concentration of 1%.
  g. Spin down at 1,000 × g for 1 minute.
  h. SpeedVac the samples until completely dry.

**CRITICAL:** Formaldehyde (CH_2_O, CD_2_O, ^13^CD_2_O) is toxic and carcinogenic. Sodium cyanoborohydride is toxic and causes severe irritation. Ammonia is corrosive and can irritate or burn the skin, eyes, and respiratory tract. Handle all in a fume hood with appropriate PPE.

***Note:*** Reconstitute peptides in Buffer A to 1 ng/μL for Evotip loading or store the dried samples at −20°C for future LC-MS analysis.

18. Experimental channel sample (Δ4 and Δ8 samples) preparation for LC-MS analysis.
  a. Add 4 μL of lysis buffer (60 mM TEAB with 0.01% DDM) to each well.
  b. Seal the plate and centrifuge at 2,000 × g for 10 min.
  c. Incubate at 95°C for 60 min in a thermal cycler.
  d. Cool the plate to 22°C–26°C.
  e. Centrifuge at 1,000 × g for 10 seconds before opening the lid.
  f. Add 1 μL of 60% acetonitrile (final concentration 12% acetonitrile in 5 μL total volume).
  g. Seal the plate, centrifuge at 1,000 × g for 10 seconds.
  h. Incubate at 75°C for 60 min in a thermal cycler, then cool.
  i. LysC digestion:
    i. Dilute LysC in 60 mM TEAB with 12% acetonitrile to a concentration of 4 ng/μL.
    ii. Add 1 μL of diluted LysC (4 ng total) to each well.
    iii. Seal the plate and centrifuge at 1,000 × g for 10 seconds.
    iv. Incubate at 37°C for 3 h in a thermal cycler.
  j. Trypsin digestion:
    i. Dilute trypsin in 60 mM TEAB with 12% acetonitrile to a concentration of 4 ng/μL.
    ii. Add 1.5 μL of diluted trypsin (6 ng total) to each well (final volume 7.5 μL).
    iii. Seal the plate and centrifuge at 1,000 × g for 10 seconds.
    iv. Incubate 12 to 18 h at 37°C in a thermal cycler.
19. Experimental channel (Δ4 and Δ8 samples) sample dimethyl labeling.^10^
  a. Add 1.5% intermediate (CD_2_O) or heavy (^13^CD_2_O) Formaldehyde to the sample to achieve a final concentration of 0.15%.
  b. Add 0.23 M light (NaBH_3_CN) or heavy (NaBD_3_CN) Sodium Cyanoborohydride to achieve a final concentration of 0.023 M.
    ***Note:*** This step retrieves Δ4 and Δ8 dimethyl-labeled samples: Δ4 = intermediate (CD_2_O) + light (NaBH_3_CN); Δ8 = heavy (^13^CD_2_O) + heavy (NaBD_3_CN).
  c. Seal the plate and incubate at 22°C–26°C for 1 hour.
  d. Add 1.43% Ammonia Solution to the sample to achieve a final concentration of 0.13%.
  e. Centrifuge at 1,000 × g for 1 minute, then incubate for 2 min to quench the reaction.
  f. Add 10% TFA to achieve a final TFA concentration of 1% to acidify the peptides.
  g. Centrifuge at 1,000 × g for 1 minute to ensure sample uniformity.
20. Evotip loading for LC-MS analysis.
  a. Wash the Evotips with 20 μL of Buffer B (99.9% acetonitrile, 0.1% formic acid ) and centrifuge at 700 × g for 1 minute.
  b. Activate the Evotips by immersing them in 2-propanol for 1 minute, then centrifuge at 700 × g for 1 minute.
  c. Wash the tips with 20 μL of Buffer A (0.1% formic acid) and centrifuge at 700 × g for 1 minute.
  d. Load 25 ng of the reference peptides (Δ0) first, followed by Δ4 and Δ8 samples using the same tip, then centrifuge at 700 × g for 1 minute.
  e. Rinse the sample wells with 20 μL of Buffer A and load the rinse solution onto the tips, then centrifuge at 700 × g for 1 minute.
  f. Overlay the tips with 100 μL of Buffer A and centrifuge at 700 × g for 10 seconds.

***Note:*** The Evotips are now ready for LC-MS analysis.

**CRITICAL:** 2-Propanol is flammable; handle in a fume hood with appropriate PPE. Formic acid is corrosive; use in a fume hood with PPE and store securely away from heat.

### Mass spectrometry-based single-cell-type proteomics using DIA-PASEF

**Timing: 80 samples per day (SPDs)**

This step describes liquid chromatography and mass spectrometry workflows, including spectral library generation and data acquisition for single-cell-type proteomic profiling.

21. Perform LC-MS analysis for dimethyl-labeled tonsil cancer samples using the Evosep One LC system (Evosep) coupled to a timsTOF SCP mass spectrometer (Bruker) (Troubleshooting).
  a. Generate a spectral library using five DDA-PASEF single-shot acquisitions from a 50-ng bulk reference peptide.
  b. Use the Whisper40 SPD method on the Evosep One system. Mass spectrometer settings for experimental sample runs are as follows^11^^,^^12^:
    i. Acquisition method: Optimized DIA-PASEF.
    ii. DIA-PASEF scans: 8 scans with variable width.
    iii. Ion mobility windows per scan: 2 windows.
    iv. m/z range: 300–1200.
    v. Ion mobility range: 0.7–1.3 V/cm^2^/s.
    vi. Mass spectrometer mode: High sensitivity.
    vii. Accumulation & ramp time: 100 ms.
    viii. Capillary voltage: 1400 V.
    ix. Collision energy: Linear ramp from 20 eV at 1/K_0_ = 0.6 V/cm^2^/s to 59 eV at 1/K_0_ = 1.6 V/cm^2^/s.

***Note:*** The separation employs the Aurora Elite CSI third-generation column (IonOpticks AUR3-15075C18-CSI, with a length of 15 cm and an internal diameter of 75 μm, and a particle size of 1.7 μm; Key resources table) maintained at 50°C within the CaptiveSpray ion source (Bruker).

***Note:*** mDIA employs the 40 SPD MS method. However, since each sample contains a reference channel and two experimental groups, the total throughput effectively reaches 80 SPD.

***Alternatives:*** This protocol describes the timsTOF SCP, but other high-end instruments, such as the Orbitrap Astral mass spectrometer (Thermo Fisher Scientific), may provide similar or even improved performance.^1^

### MS data analysis for spatial proteomic integration

**Timing: 5 h**

This step focuses on processing the raw mass spectrometry data to generate proteomic profiles for downstream correlation with the imaging-based spatial maps.

22. Spectral library generation.
  a. Search spectra using FragPipe v18.0, incorporating MSFragger v.3.5, Philosopher v.4.4.0, and EasyPQP v.0.1.32.
  b. Perform searches against the human FASTA reference file (2023, UP000005640_9606; Document S2).^9^
  c. Employ the DIA_SpecLib_Quant workflow with standard settings, except for:
    i. Fixed modifications: N-terminal and lysine mass shifts of 28.0313 Da.
    ii. Variable modification: Methionine oxidation.
    iii. Carbamidomethylation: Not included.
    iv. Missed cleavages allowed: 1.
    v. Precursor charge range: 2–4.
    vi. Peptide mass range: 300–1800 Da.
    vii. Peptide length: 7–30 amino acids.

***Note:*** Exclude carbamidomethylation because we do not perform alkylation.

**Figure 3:**
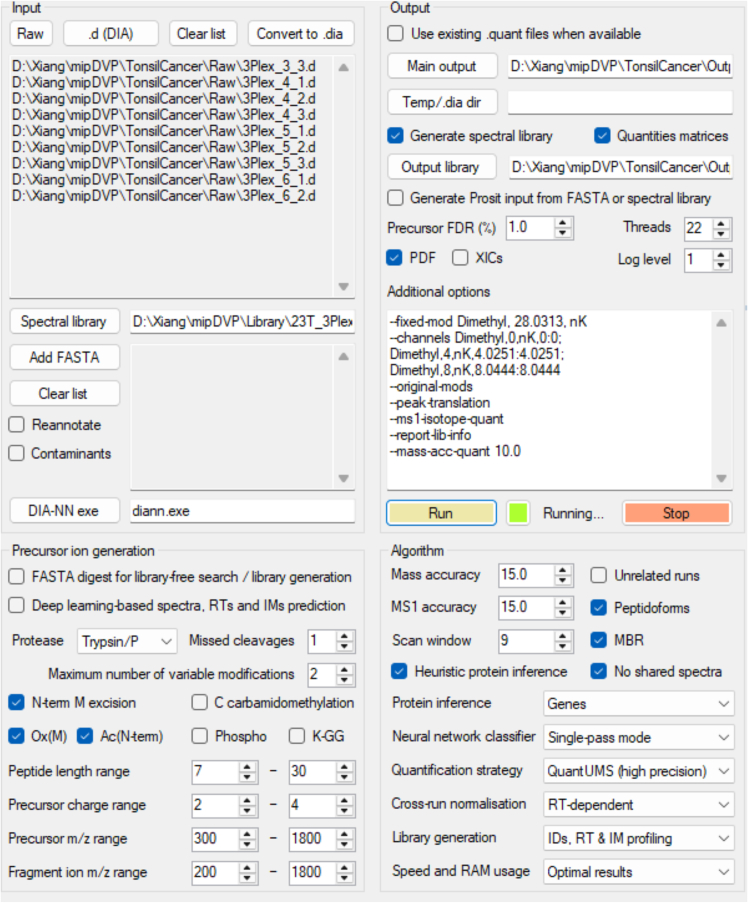
Settings in DIA-NN for mDIA experiments Screenshot of the DIA-NN (v.1.8.1) interface configured for dimethyl-labeled proteomic data analysis.

23. Analyze DIA raw files using DIA-NN v.1.8.1, utilizing the generated library^8^ (Figure 3; Table 2).

**Table 2:** DIA-NN search parameters

Parameter	Setting
Protease	Trypsin/P
Missed cleavages	1
Post-translational modifications	N-terminal methionine excision Methionine oxidation N-terminal acetylation Max of 2 variable modifications
Precursor FDR	1%
Mass accuracy	15 ppm
MS1 accuracy	15 ppm
Scan window	9
Protein inference	Genes
Neural network classifier	Single-pass mode
Quantification strategy	Robust LC (high precision)
Cross-run normalization	RT-dependent
Library generation	IDs, RT, and IM profiling
Additional parameters	Use isotopologues Match between runs (MBR) Heuristic protein inference No shared spectra
DIA-NN command line settings	--fixed-mod Dimethyl, 28.0313, nK --channels Dimethyl, 0, nK, 0:0; Dimethyl, 4, nK, 4.0251:4.0251; Dimethyl, 8, nK, 8.0444:8.0444 --original-mods --peak-translation --ms1-isotope-quant --report-lib-info --mass-acc-quant 10.0
24. Post-processing and filtering.
  a. Implement the processed DIA-NN output report table undergoes additional analysis using the ‘RefQuant’ package in Python. Filter the RefQuant output for ‘Lib.PG.Q.Value’ < 0.01, ‘Q.value’ < 0.01 and ‘Channel.Q.Value’ < 0.15.^10^
  b. Consolidate the data into protein groups using the MaxLFQ algorithm in the R package ‘iq’^13^:

library(iq)

sp_norm_data <- iq::preprocess(quant_table = sp_tab,

primary_id = "protein",

secondary_id = c("ion"),

sample_id = "experiment",

intensity_col = "quant",

median_normalization = FALSE)

sp_protein_list <- iq::create_protein_list(sp_norm_data)

sp_result <- iq::create_protein_table(sp_protein_list)

write.table(cbind(Protein = rownames(sp_result$estimate),

sp_result$estimate,

annotation = sp_result$annotation),

"result.txt", sep = "\t", row.names = FALSE)

## Expected outcomes

**Figure 4:**
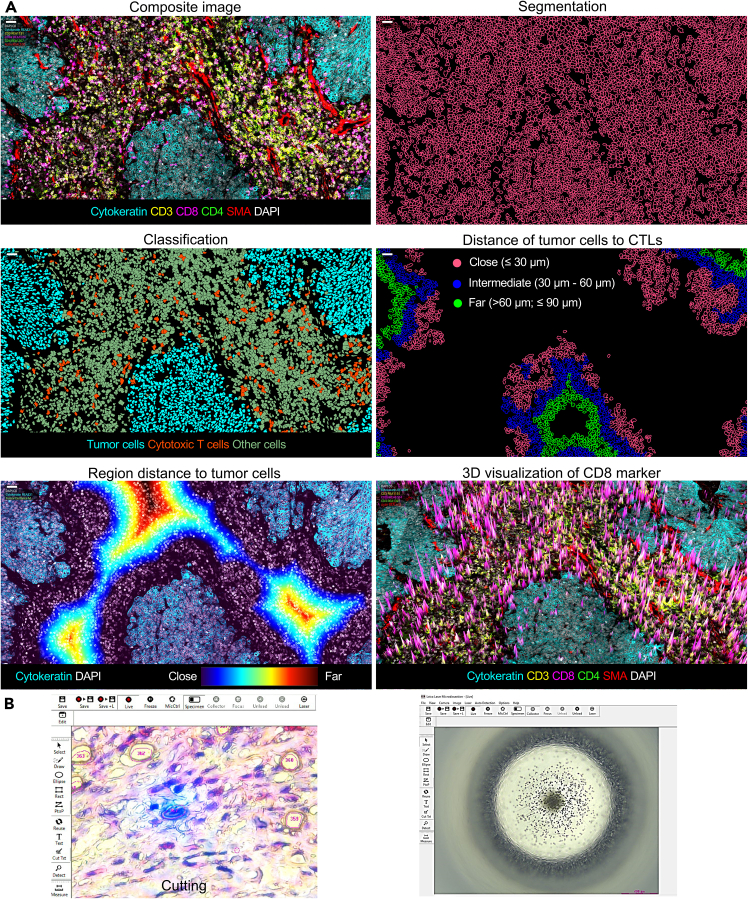
Workflow for spatial analysis and cell isolation (A) Overview of tumor-immune spatial mapping in tonsil carcinoma. The composite multiplex immunofluorescence image (top left) shows a tonsil carcinoma tissue section stained for cytokeratin (tumor cells), CD3 and CD8 (CTLs), CD4 (helper T cells), smooth muscle actin (SMA; stroma), and DAPI (nuclei). Automated segmentation (top right) identifies individual cells, with classification masks (middle left) distinguishing tumor cells (cyan) and CTLs (orange). Categorize tumor cells into three proximity zones relative to CTLs: close (≤30 μm, pink), intermediate (30-60 μm, blue), and far (60–90 μm, green; middle right). A density gradient map (lower left) visualizes tumor cell distances to stromal interfaces (blue: proximal, red: distal). A 3D volumetric rendering (lower right) depicts CD8+ T cell clusters (magenta isosurfaces) localized near stromal-tumor boundaries. Scale bars: 25 μm. (B) Automated laser microdissection workflow. Spatial classification masks recognize cellular contours and precisely excise them. Collect microdissected single cells via gravity into a 384-well plate (right) for downstream proteomic analysis. Panel B is adapted and modified from Zheng et al. under the terms of the CC-BY license.^1^

Using 22-plex imaging reveals spatial niches in tonsil carcinoma, enabling stratification of tumor cells (cytokeratin+) into three proximity zones relative to CTLs (CD3+CD8+): close (≤30 μm), intermediate (30-60 μm), and far (60-90 μm). This stratification reveals potential immune evasion mechanisms in tumor cells distal to CTLs and defines patterns of CTL infiltration into tumor islands. Three-dimensional rendering of CD8+ T cell clusters maps CTL hotspots near stromal interfaces, highlighting regions of potential active immune surveillance (Figure 4A). This spatial context enables automated laser microdissection of distinct cell populations or subpopulations, guided by exported classification masks.

The integration of spatial mapping with automated laser microdissection enables researchers to isolate spatially resolved stromal cells and tumor populations (approximately 37,000 μm^2^ surface area) for downstream proteomic profiling. This workflow links spatial immune-tumor interactions to functional protein signatures and identifies therapeutic targets relevant to precision oncology (Figure 4B).

**Figure 5:**
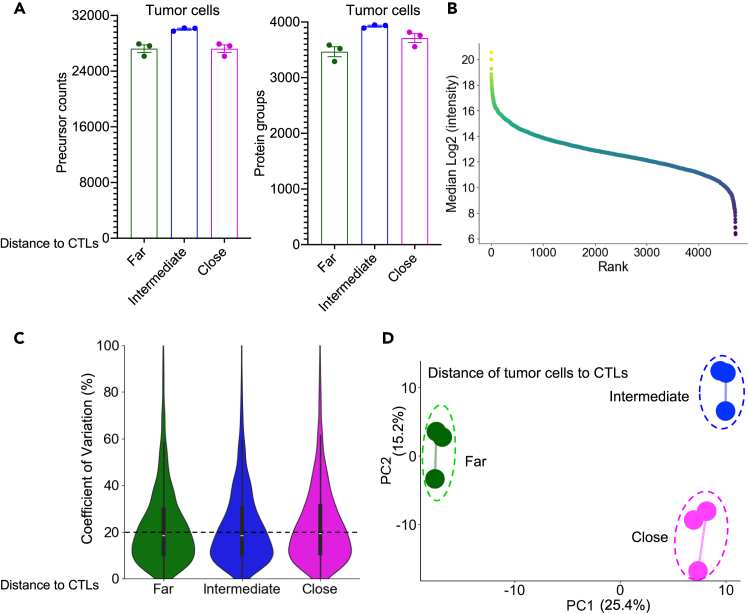
Proteomic profiling of spatially resolved tumor cell populations (A) Quantification of peptide precursors and protein groups across technical replicates (n=3) for tumor cells stratified by proximity to cytotoxic T lymphocytes (CTLs): far (60-90 μm), intermediate (30-60 μm), and close (≤30 μm). The data are presented as mean ± SEM. (B) Protein abundance rank plot summarizing cumulative identification of >4,900 protein groups across all tumor cell populations. (C) Represent technical reproducibility of proteomic data as coefficients of variation (CVs, %) for quantified protein groups. (D) Principal component analysis (PCA) of tumor cell proteomes, illustrating distinct clustering of far-proximity populations compared to intermediate and close groups. Panel D is adapted and modified from Zheng et al. under the terms of the CC-BY license.^1^

The protocol uses the timsTOF SCP platform to analyze dimethyl-labeled samples. Tumor cells far from CTLs yield approximately 27,000 peptide precursors and 3,500 protein groups, while intermediate- and close-proximity tumor cells generate around 31,000 precursors (3,900 protein groups) and 27,000 precursors (3,700 protein groups), respectively (Figure 5A). Across replicates, researchers can expect cumulative detection of approximately 4,900 protein groups (Figure 5B), with median technical reproducibility (coefficient of variation) at ≤20% (Figure 5C). Principal component analysis (PCA) of these datasets distinguishes tumor cells based on their proximity to CTLs, with the distant group clustering apart from close and intermediate groups (Figure 5D). This confirms the protocol’s ability to resolve single-cell-type niche-specific signatures.

Linking immune-tumor distances to protein signatures (e.g., metabolic regulators, immune checkpoints), helps identify microenvironment-dependent therapeutic targets.^14^^,^^15^^,^^16^^,^^17^ This workflow standardizes the integration of spatial histology with proteomic heterogeneity, advancing studies of immune evasion and precision oncology strategies.

## Limitations

Even with advanced MS techniques (e.g., diaPASEF on timsTOF SCP), the protocol is constrained by sample input, instrument sensitivity, and potential peptide loss during low-volume handling steps when assessing proteome coverage per cell type. Variability results from sample preparation, including incomplete laser microdissection or evaporation during high-temperature incubation. Currently optimized for tonsil cancer microenvironments, adapting it to other cancer types or tissues may require adjustments in antibody panels, laser settings, or protease digestion. This method captures a static snapshot of the tumor microenvironment, so it benefits from complementary functional assays to infer dynamic interactions.

## Troubleshooting

### Problem 1: Low antibody staining intensity (related to steps 4 and 6)

Address issues with low fluorescence signal caused by suboptimal antibody binding or insufficient antigen retrieval.

### Potential solutions

• Verify antibody expiration dates and storage conditions and validate antibody activity using control tissues.
• Extend xylene/ethanol deparaffinization cycles if residual paraffin remains.
• Extend heat-induced antigen retrieval time to 60 min and ensure slides are fully submerged during heat-induced retrieval.
• In the case of decreased signal-to-noise ratio due to increased excessive background fluorescence, consider recalibrating MACSima Lightbox 3 using the ‘Hardware Check’ module.

### Problem 2: Unsatisfactory cellular segmentation (related to step 10)

Resolve issues with cell segmentation, such as overlapping boundaries or missegmented cells, during image analysis.

### Potential solutions

• Adjust ‘Detection sensitivity’ (increase for sparse cells; decrease for dense regions) (Figure 2A).
• Tune ‘Separation force’ (higher values improve splitting of overlapping cells) (Figure 2A).
• Replace ‘Auto’ mode with manual selection of cytoplasmic markers as reference channels to refine boundary detection (Figure 2A).
• Train a custom segmentation model using tools like Cellpose or DeepCell on representative images, then import precomputed masks into MACS iQ View software for downstream analysis.^5^^,^^18^
• Manually validate segmentation accuracy on multiple regions and iteratively refine parameters.

### Problem 3: Misaligned masks for laser microdissection (related to step 13)

Fix problems with mask alignment that may lead to imprecise laser microdissection of target cells.

### Potential solutions

• Recalibrate alignment using BIAS software with three reference points (these points could be natural features of the tissue, such as blood vessels, nuclei, or other morphological structures that are easily identifiable).
• Verify mask coordinates in XML files and cross-check with DAPI/H&E overlay in Leica LMD software.

### Problem 4: Low yield or sample loss during microdissection (related to step 14)

Troubleshoot reduced sample recovery or damaged membrane slides that impact microdissection yield.

### Potential solutions

• Handle PEN slides with care to avoid damaging the membrane, as any damage can trap liquid underneath, negatively affecting microdissection.
• Use fresh PEN slides and avoid prolonged UV exposure during pretreatment.
• Reduce laser power to 30% and increase pulse frequency to 3,000 Hz.

### Problem 5: Low protein identification from MS data (related to step 21)

Resolve issues with insufficient protein identification, such as poor labeling efficiency, sample evaporation, or MS calibration errors.

### Potential solutions

• For high sample evaporation in 384-well plates, use adhesive PCR foil sheets in duplicate and centrifuge the plates before sealing. Avoid using the outermost wells and include buffer-only controls to monitor evaporation.
• For suboptimal MS sensitivity, check the cleanliness of the ion source and recalibrate the mass spectrometer as needed.
• For potential low labeling efficiency, check labeling by analyzing 1–10 ng of labeled samples in DDA-PASEF mode on a timsTOF SCP and assess efficiency using MaxQuant to confirm proper dimethyl incorporation.^10^^,^^19^ High efficiency (>99%) ensures accurate quantification; if efficiency is low, adjust labeling conditions, reagent concentrations, or reaction times.
• For poor spectral library coverage, fractionate reference samples and combine predicted libraries such as AlphaPept Deep with experimental libraries.^20^ In FragPipe, ensure fixed dimethyl modifications of 28.0313 Da are applied to ‘N-termini and lysines’.^10^

## Resource availability

### Lead contact

Further information and requests for resources and reagents should be directed to and will be fulfilled by the lead contact, Xiang Zheng (xiang.zheng@cpr.ku.dk).

### Technical contact

Technical questions on executing this protocol should be directed to and will be answered by the technical contact, Xiang Zheng (xiang.zheng@cpr.ku.dk).

### Materials availability

This study did not generate new unique reagents.

### Data and code availability

The accession number for the mass spectrometry proteomics data reported in this paper is PRIDE: PXD057118.^21^ The accession number for the multiplexed imaging data reported in this paper is BioImage Archive: S-BIAD1116.^22^ This study does not report any original code.

## Acknowledgments

The work was supported by the Novo Nordisk Foundation (NNF14CC0001 and NNF15CC0001) and the Max Planck Society for the Advancement of Science.

## Author contributions

Conceptualization, X.Z., A.M., and M.M.; methodology, X.Z., A.M., and M.M.; data curation, X.Z.; formal analysis, X.Z.; visualization, X.Z.; funding acquisition, M.M.; writing – original draft, X.Z. All authors contributed to the review and editing of the manuscript.

## Declaration of interests

M.M. is an indirect investor in Evosep Biosystems and OmicVision Biosciences. A.M. is a co-founder and chief scientific officer of OmicVision Biosciences.

## Declaration of generative AI and AI-assisted technologies in the writing process

During the preparation of this work, the authors used ChatGPT 4o and DeepSeek v.3 in order to help edit the manuscript. After using this tool/service, the authors reviewed and edited the content as needed and take full responsibility for the content of the publication.
